# Characterizing Isozymes of Chlorite Dismutase for Water Treatment

**DOI:** 10.3389/fmicb.2017.02423

**Published:** 2017-12-12

**Authors:** Kellen C. Mobilia, Justin M. Hutchison, Julie L. Zilles

**Affiliations:** Department of Civil Environmental Engineering, University of Illinois at Urbana–Champaign, Urbana, IL, United States

**Keywords:** biocatalyst, catalytic inactivation, chlorite, drinking water, perchlorate

## Abstract

This work investigated the potential for biocatalytic degradation of micropollutants, focusing on chlorine oxyanions as model contaminants, by mining biology to identify promising biocatalysts. Existing isozymes of chlorite dismutase (Cld) were characterized with respect to parameters relevant to this high volume, low-value product application: kinetic parameters, resistance to catalytic inactivation, and stability. Maximum reaction velocities (*V*_max_) were typically on the order of 10^4^ μmol min^-1^ (μmol heme)^-1^. Substrate affinity (*K*_m_) values were on the order of 100 μM, except for the Cld from *Candidatus* Nitrospira defluvii (NdCld), which showed a significantly lower affinity for chlorite. NdCld also had the highest susceptibility to catalytic inactivation. In contrast, the Cld from *Ideonella dechloratans* was least susceptible to catalytic inactivation, with a maximum turnover number of approximately 150,000, more than sevenfold higher than other tested isozymes. Under non-reactive conditions, Cld was quite stable, retaining over 50% of activity after 30 days, and most samples retained activity even after 90–100 days. Overall, Cld from *I. dechloratans* was the most promising candidate for environmental applications, having high affinity and activity, a relatively low propensity for catalytic inactivation, and excellent stability.

## Introduction

Micropollutants and other emerging contaminants are challenging to remediate due to their diverse chemical structures, their persistence in the environment, the toxicity of some degradation products, and their ability to cause toxic effects at low concentrations. Recent studies have proposed the removal of emerging contaminants through novel biocatalytic approaches, in which biological enzymes are used in the absence of cells as catalysts (Hutchison et al., 2013; Chen et al., 2016; Martínez et al., 2017; Yang et al., 2017). Potential advantages of this approach include targeted, complete degradation even in complex environmental matrices and at low concentrations. Some biological enzymes also provide less specific catalytic activities that may have applications in remediation, as shown for example for unspecific peroxygenases derived from fungi (Karich et al., 2017). However, environmental applications require greater stability of the biocatalysts than is typically needed in applications with high-value products, such as pharmaceutical production (Hutchison et al., 2017). This work investigated possible biocatalysts for chlorite degradation in water treatment applications.

Chlorite is regulated by the United States Environmental Protection Agency at 1 mg L^-1^ in drinking water, while the World Health Organization has set a provisional guideline of 0.7 mg L^-1^ (World Health Organization, 2005). The health effects of chlorite, similar to those of nitrite, arise primarily through oxidative damage to red blood cells (World Health Organization, 2005). Chlorite is produced in drinking water when chlorine dioxide is used as a disinfectant (Richardson and Postigo, 2012) and in industrial processes such as wood pulp processing (World Health Organization, 2005). Chlorite could also be produced from incomplete degradation of other chlorine oxyanions like chlorate and the endocrine-disruptor perchlorate. Existing treatment methods for chlorite removal require addition of ferrous iron or thiosulfate and are sensitive to pH (World Health Organization, 2005). In contrast, the enzyme chlorite dismutase (Cld) converts chlorite into chloride and molecular oxygen via an intramolecular redox reaction, not requiring any external electron donor (van Ginkel et al., 1996).

For environmental applications, in addition to kinetic parameters, the catalytic life and stability of Cld are important considerations. Multiple investigators have found that Cld has a limited catalytic life, irreversibly inactivating after a limited number of turnovers (Stenklo et al., 2001; Streit and DuBois, 2008; Hofbauer et al., 2014a). Despite previous work with several Cld isozymes (Schaffner et al., 2015), to our knowledge the maximum turnover number has only been quantified for two Clds (Streit and DuBois, 2008; Celis et al., 2015). Catalytic inactivation of Cld is believed to be due to the occasional escape of a hypochlorite intermediate from the active site of the enzyme, resulting in irreversible oxidative damage (Hofbauer et al., 2014a). Furthermore, when the stability of perchlorate-reducing biocatalysts was examined under non-reactive conditions, chlorite was the first intermediate to accumulate, suggesting that Cld has the shorter shelf life of the two enzymes (Hutchison et al., 2013). These previous observations on catalytic inactivation and shelf life suggest that Cld would be the limiting component in biocatalytic treatment of perchlorate and chlorate, in addition to being the only biocatalyst required for chlorite removal.

We hypothesize that isozymes of Cld will vary in characteristics relevant for drinking water treatment. To investigate the potential for biocatalytic chlorite degradation, we characterized several Cld isozymes, in essence mining biology for the most suitable enzyme for removal of environmental contaminants. We assembled a library of seven Cld isozymes and quantified their performance under comparable conditions. We targeted three characteristics of Cld relevant to large-scale application: kinetic parameters in soluble protein fractions, catalytic inactivation, and shelf life. The results presented here demonstrate favorable activity and stability in soluble protein fractions as well as significant variation in catalytic life, affecting which Cld isozymes would be best suited for water treatment and other environmental applications.

## Materials and Methods

### Strains and Plasmids

Native and recombinant *cld*-expressing strains used in this work are described in **Table 1**. Recombinant plasmids were transferred by electroporation into *Escherichia coli* BL21 (DE3) using a MicroPulser^TM^ Electroporator (BioRad, Hercules, CA, United States) and 0.1 cm electroporation cuvettes, following the manufacturer’s instructions. All the expression plasmids used in this work utilized the Bacteriophage T7 Promoter system.

**Table 1:** Bacterial strains and plasmids.

ATCC No.	Strain name	Abbreviation	Source	Reference
BAA-1730	*Magnetospirillum bellicus* VDY	Mb	ATCC	Thrash et al., 2010
BAA-777	*Azospira oryza*e GR-1	AoGR	ATCC	van Ginkel et al., 1996
BAA-1848	*Dechloromonas aromatica* RCB	Da	ATCC	Streit and DuBois, 2008
BAA-33	*Azospira oryzae* PS	AoPS	ATCC	

**Plasmid**	**Origin of *cld* gene**	**Abbreviation**	**Source**	**Reference**

pET51b-AoPScld	*Azospira oryzae* PS ^a^	rAoPS		This work
pET21b-Ndcld	*Candidatus* Nitrospira defluvii	rNd	Dr. Daims	Maixner et al., 2008
pET21b-Nwcld	*Nitrobacter winogradskyi*	rNw	Dr. Daims	Mlynek et al., 2011
pET3a-Idcld	*Ideonella dechloratans*	rId	Dr. Rova	Danielsson Thorell et al., 2004
TTHA1485	*Thermus thermophilus* ^b^	rTt	RIKEN BRC	Ebihara et al., 2005

^a^*The recombinant version of AoPS Cld was used only for purification; it was not tested in soluble protein fraction.*

^b^*Expression of Cld from the *Thermus thermophilus* plasmid was insufficient for a full characterization of the enzyme*.

### Production of Soluble Protein Fractions Containing Cld

Native bacterial strains were grown on a modified mineral media as described (Hutchison et al., 2013), except that AlK(SO_4_)_2_-12H_2_O and H_3_BO_3_ were supplemented to a final concentration of 0.1 mg L^-1^ and yeast extract was omitted for *M. bellicus* VDY. Sodium chlorate was used as an electron acceptor instead of sodium perchlorate for all strains except *A. oryzae* PS, because of a prior report that *Magnetospirillum bellicus VDY* transiently accumulates chlorate when reducing perchlorate (Thrash et al., 2010). The headspace was 80% N_2_/20% CO_2_. Growth was carried out at 30°C with constant shaking at 150 rpm until the optical density at 600 nm (OD_600_) was between 0.5 and 0.6.

Plasmids were maintained by including ampicillin or carbenicillin to a final concentration of 50 μg mL^-1^. Production cultures of 500 mL were grown in Lennox Luria Broth media until the OD_600_ was approximately 1.0. At that time, hemin (Fisher Scientific, Hampton, NH, United States) was added to a final concentration of 0.5 mg mL^-1^. Expression was induced with 0.4 mM of isopropyl β-D-1-thiogalactopyranoside (Research Products International, Mount Prospect, IL, United States). Cultures were then allowed to express overnight (∼18 h) at 37°C with shaking at 175 rpm.

The methods to generate soluble protein fractions containing Cld were as previously described (Hutchison et al., 2013). In brief, cells were harvested by centrifugation, lysed by sonication, and fractionated by ultracentrifugation at 140,000 × *g* for 1 h at 4°C. After ultracentrifugation, the middle fraction (soluble protein fraction) was extracted using a glass pipette. Glycerol was added to a concentration of 10% v/v and aliquots were stored at -80°C.

### Cloning and Purification of Cld

The *cld* gene was amplified from *A. oryzae* PS without a signal peptide using these primers: forward (5′-TCTGG ATCCGCAGCAGGCAATGCAACCC) and reverse (5′-GATGTCGACTTAATCGGCTAGCGCCTTG). The amplified product was cloned into *E. coli* BL21 (DE3) using SalI and BamHI (New England Biolabs, Ipswich, MA, United States) digests with standard molecular biology techniques (Sambrook and Russell, 2001). Protein expression and preparation of soluble protein fractions were as described in previous sections. Cld was purified from the soluble protein fraction using Strep-Tactin Sepharose (IBA, Göttingen, Germany) in a gravity flow column following the manufacturer’s instructions, without EDTA. Protein purification was confirmed with SDS–PAGE and Coomassie staining.

### Cld Quantification Methods

#### Protein Assay

Total protein was quantified with the bicinchoninic acid protein assay kit (Thermo Fisher Scientific, Waltham, MA, United States) in 96-well plates. After incubation, the absorbance at 526 nm was measured with a SpectraMAX 384 Plus Microplate Reader (Molecular Devices, Sunnyvale, CA, United States).

#### Total Heme Assay

Total heme was measured as an estimate of active Cld concentration using the pyridine hemochrome assay developed by Berry and Trumpower (). Aliquots of soluble protein fractions were oxidized in a buffer containing 20% pyridine, 100 mM NaOH, and 0.3 mM K_3_Fe(CN)_6_. After recording the absorbance spectrum, the solution was reduced by addition of 10 μL of freshly prepared sodium dithionite stock (0.5 mg μL^-1^) and mixed by inversion. The reduced spectrum was recorded twice. If there was a difference between the two reduced spectra, another 10 μL of dithionite was added to ensure the solution was completely reduced before recording the final spectrum. The heme concentration was calculated from the difference between the final reduced spectrum and the oxidized spectrum, applying the dual wavelength extinction coefficient of 24 mM^-1^ cm^-1^ at either 556 and 540 nm, for the recombinant Clds, or 550 and 535 nm, for the native Clds.

### Cld Activity Assays

The kinetics of chlorite decomposition were monitored by quantifying product formation with an ion selective electrode (ISE) for chloride (Phoenix, Houston, TX, United States), similar to experiments in whole cells performed by Xu and Logan (2003). Data were collected at 0.5 s intervals with a National Instruments (Santa Clara, CA, United States) USB-6008 analog input module and a custom MATLAB data collection program. The probe was calibrated daily using a nine-point standard curve ranging from 1 to 1,000 ppm.

Reactions were carried out in 25 mL glass bottles that were continuously stirred at room temperature (21–23°C). The 10 mL reaction mixtures contained 0.0 to 5.0 mM sodium chlorite (Amresco, Solon, OH, United States) (prepared daily) in 10 mM phosphate buffer pH 7.0. Reactions were initiated by the addition of 10–20 μL of soluble protein fraction. As a control, the probe response to soluble protein fraction in the absence of chlorite was recorded. Typically the soluble protein fraction accounted for a response of 0.2 ppm or less. At least three independently grown and processed replicates were tested for each isozyme. The initial reaction rates were determined using a least squares fit to the linear portion of the reaction curve in OriginLab 9.1. Initial rates from at least 10 chlorite concentrations were then plotted against initial substrate concentration to generate a Michaelis–Menten plot. The maximum reaction velocity (*V*_max_) and half-saturation constant (*K*_m_) were determined by utilizing a least squares estimation of the data to the Michaelis–Menten equation (Hutchison and Zilles, 2015) in MATLAB. As a negative control for the recombinant Clds, the expression strain with an empty pET51b(+) plasmid was grown under similar conditions with and without the added hemin and assayed for Cld activity.

### Inactivation Assays

Catalytic inactivation of the Clds was quantified using two methods: activity decay and heme bleaching. In both cases, heme quantification was used as an estimate of Cld concentration in soluble protein fractions and results were reported using the maximum theoretical turnover number (MTTN). Cld isozymes were compared via one-way ANOVA analysis using Tukey’s pairwise comparison at a significance level of 95% in OriginLab 9.1. Activity decay was compared to heme bleaching with an unbalanced two-factor ANOVA (*p* < 0.05).

#### Activity Decay

Catalytic inactivation was quantified with the activity decay method, as described by Streit and DuBois (2008). Soluble protein fractions were exposed to chlorite concentrations between 0 and 200 mM at room temperature until bubble formation ceased. Substrates and products were removed through three cycles of buffer exchange with a spin filter (Amicon, 10 kD Cutoff, EMD Millipore, Hayward, CA, United States). Following the final exchange, the solution volume was adjusted to match the original volume of the soluble protein fraction and the protein concentration was quantified. Activities were measured with the ISE chloride probe assay. An identically handled sample that was not exposed to chlorite served as the reference activity. The fraction of remaining activity was plotted against the molar ratio of chlorite to heme to determine the MTTN.

#### Heme Bleaching

Catalytic inactivation was also quantified based on the extent of heme bleaching, by following a prominent Soret adsorption in the 400 nm range that is characteristic of porphyrins and other heme binding proteins (Streit and DuBois, 2008; Mayfield et al., 2013; Celis et al., 2015). Aliquots of soluble protein fraction (25 μL) were incubated with increasing concentrations of chlorite from a fresh 1.0 M stock solution. The total volume was adjusted to 1 mL with 10 mm phosphate buffer at pH 7.0. After the formation of bubbles ceased, the samples were briefly centrifuged to eliminate the bubbles, and their ultraviolet and visible spectra were recorded. The absorbance peak was calculated by manually subtracting a linear baseline, then the peak height was plotted against the number of turnovers per heme. Complete conversion of chlorite to chloride was assumed. Similarly to monitoring of activity decay, a line was fit to the initial linear region of the curve, and the *x*-intercept corresponded to the MTTN.

### Shelf Life Experiments

The long-term stability of the various Clds was determined by tracking the decrease in *V*_max_ of samples stored at 4 and at 22°C. Following glycerol addition, the soluble protein fraction was aliquoted into 1.5 mL microcentrifuge tubes and sealed with parafilm. For each timepoint, chlorite decomposition was measured at 1.5 and 3.0 mM sodium chlorite in duplicate from a single aliquot. Assays were conducted at room temperature, which typically fluctuated between 21 and 23°C, with one data point taken at 25°C. Sample activities were adjusted to relative activities at 22°C using the Arrhenius equation (Hutchison and Zilles, 2015). Changes in activity were normalized to the activities at 1.5 and 3.0 mM measured on day zero.

### Statistical Analysis

Data are reported as averages with standard deviation. ANOVA analysis was performed in OriginLab 9.1 with differences reported at a significance level of 95%. Cld isozymes were compared via one-way ANOVA analysis using Tukey’s pairwise comparison. Activity decay was compared to heme bleaching with an unbalanced two-factor ANOVA.

## Results

### Kinetic Parameters

Kinetic parameters *K*_m_ and *V*_max_ were measured for soluble protein fractions containing each of the seven Cld isozymes and are given in **Table 2**. The most striking observation was that rNdCld has a relatively high *K*_m_. The remaining six Clds all had lower, and similar, values for *K*_m_. For comparison, purified rAoPS Cld had a *K*_m_ of 180 μM. Attempts to quantify Cld in the soluble protein fractions using Western blots were unsuccessful due to different affinities for the isozymes. Throughout this work, *V*_max_ values were therefore normalized to total heme rather than Cld concentration. This approach has been used previously to quantify active, purified Cld, accounting for incomplete incorporation of heme with an approximately 1:1 molar ratio of heme to active Cld protein (Streit and DuBois, 2008). In the current work, this approach also accounts for any differences in expression of Cld, assuming that non-Cld heme is either negligible or constant in the soluble protein fractions. *V*_max_ values and catalytic efficiencies were relatively similar across the seven Clds (**Table 2**). The only statistically significant difference in catalytic efficiency was between rNwCld and MbCld. Using the same normalization method, purified rAoPS Cld had a *V*_max_ of 7.2^∗^10^4^ μmol min^-1^ μmol_heme_^-1^. Cld activity was below detection in the expression strain with an empty vector.

**Table 2:** Michaelis–Menten parameters for Cld isozymes in soluble protein fractions.

Origin	Replicates	*K*_m_ (μM)	Published *K*_m_ (μM) ^a^	Replicates	*V*_max_ (μmol min^-1^ μmol_heme_^-1^) ^b^	*k*_cat_/*K*_m_ (M^-1^ s^-1^)
AoGRCld	7	330 ± 110 ^c^	170	3	2.8 ^∗^ 10^4^ ± 1.5 ^∗^ 10^4^	1.4 ^∗^ 10^6^
rIdCld	6	320 ± 160	260	3	4.6 ^∗^ 10^4^ ± 2.8 ^∗^ 10^4^	2.4 ^∗^ 10^6^
rNdCld	5	2500 ± 470	15,800 and 58 ± 37	3	19 ^∗^ 10^4^ ± 4.1 ^∗^ 10^4^	1.3 ^∗^ 10^6^
rNwCld	6	380 ± 150	90	3	0.95 ^∗^ 10^4^ ± 0.26 ^∗^ 10^4^	0.42 ^∗^ 10^6^
AoPSCld	3	360 ± 54	N/A	3	5.0 ^∗^ 10^4^ ± 1.7 ^∗^ 10^4^	2.3 ^∗^ 10^6^
DaCld	5	290 ± 170	215 ± 25	3	1.9 ^∗^ 10^4^ ± 0.52 ^∗^ 10^4^	1.1 ^∗^ 10^6^
MbCld	7	360 ± 210	N/A	3	7.9 ^∗^ 10^4^ ± 3.9 ^∗^ 10^4^	3.7 ^∗^ 10^6^

^a^*References for published *K*_*m*_ values are as follows: AoGRCld (van Ginkel et al., 1996), native IdCld (Stenklo et al., 2001), rNdCld 15,800 (Maixner et al., 2008) and 58 (Kostan et al., 2010), rNwCld (Mlynek et al., 2011), and rDaCld (Streit and DuBois, 2008).*

^b^* Value normalized to total heme content.*

^c^* ± reports standard deviation*.

### Catalytic Inactivation

**FIGURE 1:**
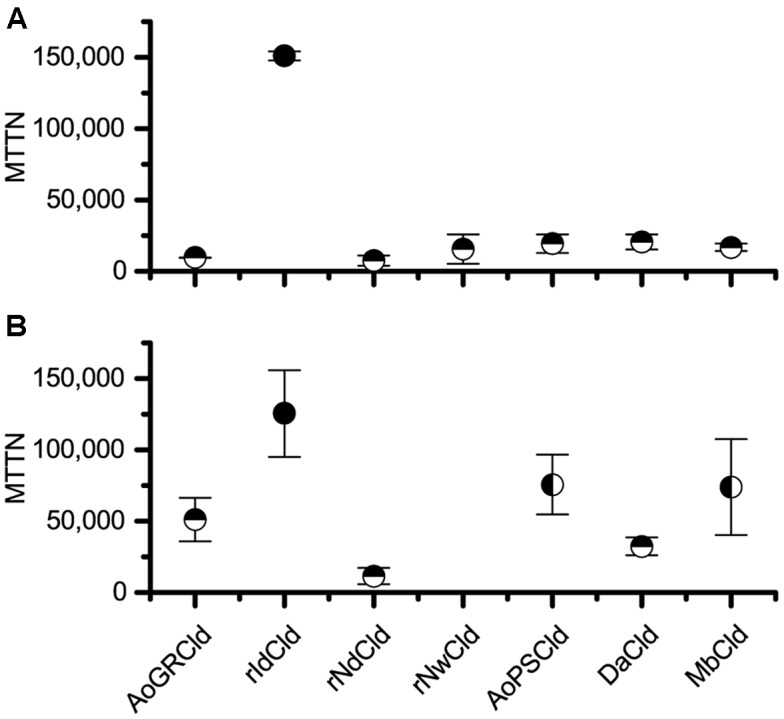
Maximum theoretical turnover number. Comparison of maximum theoretical turnover calculated via activity decay **(A)** and heme bleaching **(B)**. The average of three biological replicates is shown for each isozyme, with error bars showing standard deviation. Horizontal half circles denote significant difference (*p* < 0.05) in mean as compared with rIdCld. Vertical half circles denote significant difference (*p* < 0.05) as compared to rNdCld. rNwCld was not measured by the heme bleaching method because of poor expression.

Two methods were used to quantify inactivation: activity decay and heme bleaching. Comparing the seven Cld isozymes with the activity decay method, rIdCld had an MTTN of 150,000, while the others had catalytic lifespans equal to or shorter than DaCld at 21,000 turnovers (**Figure 1A**). With this method, the only statistically significant difference in MTTN was between rIdCld and all the other Clds. The trends from the heme bleaching method were generally in agreement with the results from activity decay (**Figure 1B**). The rNwCld was not tested with this method due to poor expression. With the heme bleaching method, the rIdCld again had a significantly larger MTTN of 130,000, as compared to the other isozymes. rNdCld had the lowest MTTN of 11,000; this value was significantly lower than AoPSCld and MbCld as well as rIdCld.

### Shelf Life

**FIGURE 2:**
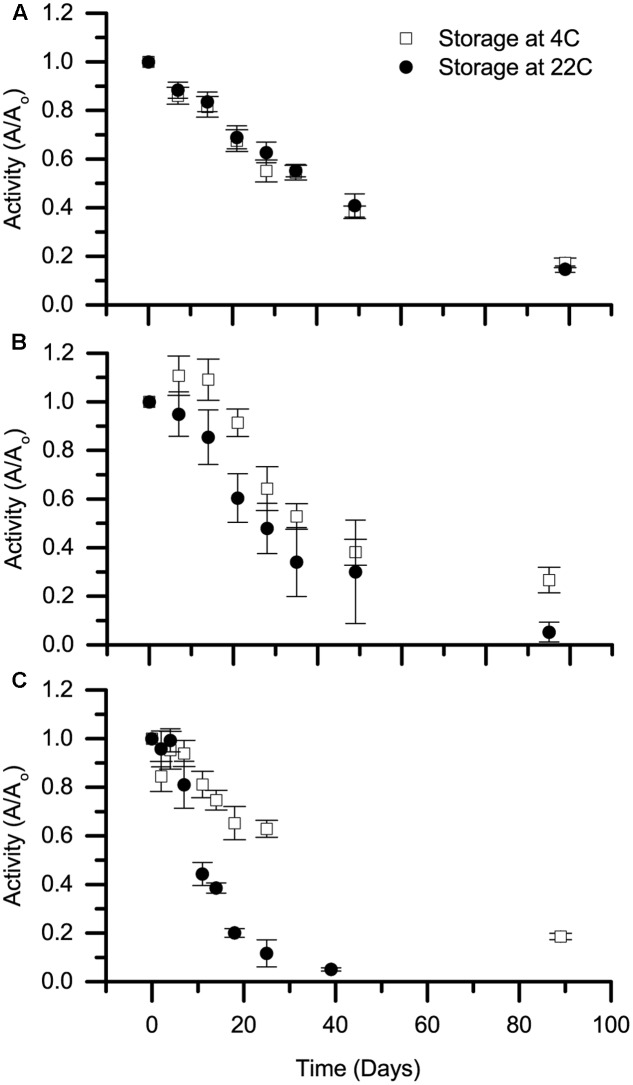
Shelf life of Cld. Stability of stored chlorite dismutase, referred to as shelf life, is presented for **(A)** soluble protein fraction containing rIdCld, **(B)** soluble protein fraction from *A. oryzae* PS, and **(C)** purified, heterologously expressed Cld from *A. oryzae* PS. Storage temperatures were 4 (open squares) and 22°C (solid circles). The average decay in activity from three biological replicates over time was normalized to day zero activity at the specified storage temperature. Error bars represent standard deviation.

A key concern for environmental applications is whether biocatalysts will have sufficient stability to be transported and stored in a cost-effective manner. The stability of selected Cld isozymes under non-reactive conditions, referred to here as shelf life, was therefore tested at 4 and 22°C. Specifically, rIdCld was tested due to its high MTTN, and AoPSCld was tested both in soluble protein fractions and in purified form. The activity was surprisingly stable (**Figure 2**). The soluble protein fractions had Cld activity even after 90–100 days.

## Discussion

Three protein families share structural similarities within the peroxidase-chlorite superfamily. The families are Cld, Cld-like, and Dyp-type peroxidases. This work focused primarily on the Cld family, as the other families exhibit slower chlorite degradation (Zamocky et al., 2015). Seven Clds were tested, including two from species not known to reduce perchlorate or chlorate (*Candidatus* Nitrospira defluvii and *Nitrobacter winogradskyi*). An expression plasmid for one Cld-like protein, from *Thermus thermophilus*, was tested, but did not show good expression or detectable activity, so it was excluded from further analysis.

The kinetics observed here from soluble protein fractions showed a general trend of being slightly less favorable than published values for purified Cld. The *K*_m_ values tended to be slightly higher, while the catalytic efficiencies (*k*_cat_/*K*_m_ ratios) tended to be slightly lower. These differences are not due to our assay procedures, since the purified rAoPSCld showed good agreement with published values. Its *K*_m_ was 180 μM, as compared to the published *K*_m_ of 170 μM for AoGRCld, and its *k*_cat_ was 930 s^-1^, as compared to the published *k*_cat_ of 1,200 s^-1^, also for AoGRCld (van Ginkel et al., 1996). Differences between previously published values and those measured here could be due to the use of soluble protein fractions, as compared to purified proteins in previous work. For large-scale treatment applications, slight improvements in kinetic parameters for purified Clds would have to be weighed against the additional costs incurred by purification.

Comparing the two methods of measuring catalytic inactivation, the MTTNs found using the heme bleaching were significantly larger. Reasonable agreement was observed between the MTTN measured here for DaCld (21,000) and the previously reported value for purified rDaCld (17,000) (Streit and DuBois, 2008), and a similar discrepancy between the two methods was previously reported (Streit and DuBois, 2008; Celis et al., 2015). The most likely explanation is that the MTTNs measured by heme bleaching are artificially high due to the inability of this method to capture non-heme damage to Cld, since inactivation is known to occur through damage to both the heme moiety and the protein backbone (Hofbauer et al., 2014a). Two other possible explanations are specific to the conditions used in this study. One is that if non-Cld heme were less susceptible to damage, the presence of non-Cld heme in the soluble protein fractions could decrease the observed heme bleaching and therefore give a higher observed MTTN in the heme bleaching assay. The other is that the small reaction volume used here in the heme bleaching assays could have resulted in some product inhibition, slowing the reaction rate. This could have resulted in incomplete chlorite degradation and again given a higher observed MTTN. In these reactions, chloride concentrations reached up to 500 mM, which is higher than the estimated inhibitory constant (*K*_I_) of 225 mM (Streit and DuBois, 2008). However, the fact that similar discrepancies between the two methods were observed in two previous studies with purified Clds and larger reaction volumes (Streit and DuBois, 2008; Celis et al., 2015) suggests that most of the observed difference is due to non-heme damage.

The rNdCld had the lowest MTTN at 7,000 turnovers. For comparison, data from another study of NdCld can be used to estimate an MTTN of 5,000 turnovers (Hofbauer et al., 2014a). In combination with its relatively high *K*_m_, this low MTTN suggests that NdCld would be a poor candidate for biocatalytic water treatment. This conclusions run counter to other studies promoting NdCld as a promising candidate for biotechnology applications (Hofbauer et al., 2014b). However, those conclusions were based on the greater thermal and conformation stability of NdCld as compared to NwCld (Hofbauer et al., 2012), and did not consider the relatively high *K*_m_ and low MTTN reported here for NdCld, which do not support its use in biocatalytic applications.

To understand the implications of these catalytic inactivation results for drinking water treatment applications, we examined two scenarios of chlorite contamination. The first scenario modeled a perchlorate treatment system as previously described (Hutchison and Zilles, 2015), with an influent concentration of 100 μg perchlorate L^-1^ and an effluent concentration of 10 μg perchlorate L^-1^. Based on the MTTN from activity decay and a Cld to total protein ratio of 0.25 ± 0.09, only 0.11% of the chlorite degradation capacity for the *A. oryzae* soluble protein fraction would be required under this scenario. *A. oryzae* soluble protein fraction was used for these calculations because it contains both perchlorate reductase and Cld.

The second scenario modeled applications requiring treatment of chlorite, where the influent and effluent chlorite concentrations are higher. This model was run for an influent of 5 mg L^-1^ (Michael et al., 1981) and an effluent concentration of 1 mg L^-1^, based on the United States Environmental Protection Agency’s Primary Drinking Water standard. In this second scenario, 7.4% of the chlorite degradation capacity was required. Alternatively, using soluble protein fraction with rIdCld and an expression ratio of 0.08 ± 0.01, the required chlorite decomposition only consumed 2.1% of Cld capacity. Catalytic inactivation of Cld is therefore unlikely to limit biocatalytic perchlorate or chlorite degradation. With development of an immobilized enzyme system, catalytic inactivation could become limiting in either scenario, but in that case the Cld dosage could be increased. It is also possible that the catalytic life of Cld could be extended further. Some mammalian peroxidases are protected from substrate-mediated inactivation through covalent linkages between the polypeptide and the porphyrin moiety (Wojciechowski et al., 2005; Jakopitsch et al., 2014). However, no Clds are known to or predicted to have this motif.

The shelf life experiments evaluate the potential storage of the biocatalysts before use. These results are especially notable considering that nothing was added to prevent growth or protease activity in these samples. The shelf life at 4°C was similar to a previous study on perchlorate reducing biocatalysts (Hutchison et al., 2013), while the 22°C shelf life observed here was longer. The discrepancy may be due to the use of temperature-controlled storage in this study. The purified rAoPSCld was the only one to completely lose activity during the test period. As the purified rAoPSCld lost activity more quickly than the same isozyme in the soluble protein fraction, we hypothesize that the soluble protein fraction contains other compounds that are responsible for the increased shelf life.

Overall, Cld from *Ideonella dechloratans* exhibited the best properties for water treatment applications: low *K*_m_, high *V*_max_, low susceptibility to catalytic inactivation, and a long shelf life. Based on the turnover numbers observed here, catalytic inactivation could become an important design consideration if the biocatalysts were immobilized for reuse in perchlorate or chlorite removal. Continuing to identify or develop Clds with greater resistance to inactivation would therefore aid in development of this biocatalytic technology. Overall, this work supports the feasibility of biocatalytic degradation of environmental contaminants such as chlorite and perchlorate.

## Author Contributions

KM, JH, and JZ contributed to the conception and design of the work and production and revision of the manuscript. KM and JH collected and interpreted data. All authors read and approved the final submission.

## Conflict of Interest Statement

The authors declare that the research was conducted in the absence of any commercial or financial relationships that could be construed as a potential conflict of interest.
